# Vascular anomalies in patients with growth hormone-secreting pituitary adenomas: illustrative case report and systematic review of the literature

**DOI:** 10.1007/s11102-022-01291-3

**Published:** 2022-12-12

**Authors:** Dorothee Cäcilia Spille, Elena Vorona, Michael P. Catalino, Gilles Reuter, Albert Beckers, Markus Holling, Arianna Fava, Walter Stummer, Edward R. Laws, Eric Suero-Molina

**Affiliations:** 1 Department of Neurosurgery, University Hospital of Münster, Albert-Schweitzer-Campus 1, A1, 48149 Münster, Germany; 2 Department of Medicine B of Gastroenterology, Hepatology, Endocrinology and Clinical Infectiology Division for Endocrinology and Diabetes, 48149 Münster, Germany; 3 The University of Texas, MD Anderson Cancer Center, Houston, TX USA; 4 Department of Neurosurgery, University Hospital of Liège, Liège, Belgium; 5 Centre Hospitalier Universitaire de Liège, Liège, Belgium; 6 Department of Neurosurgery, I.R.C.C.S. Neuromed, Pozzilli, IS Italy; 7 Department of Neurosurgery, Brigham and Women’s Hospital, Boston, MA USA

**Keywords:** Pituitary, Adenoma, Acromegaly, Endothelial dysfunction, Growth hormone

## Abstract

**Purpose:**

Endonasal resection is the first-line treatment for patients harboring growth hormone (GH)-secreting pituitary adenomas. The complexity of the parasellar neurovascular structures makes pre-operative diagnostic imaging essential to understanding the anatomy of this region. We aimed to describe vascular anomalies in acromegalic patients and emphasize their relevance for surgery and preoperative planning.

**Methods:**

A systematic review following the PRISMA statement was performed in July 2021.

**Results:**

Thirty-three studies were evaluated. Elevated GH and insulin-like growth factor-1 (IGF-1) levels are linked to the occurrence of cardiovascular risk factors. This is attributed to endothelial dysfunction, mainly caused by changes in flow-mediated dilatation (FMD), which is probably the main cause of vascular anomalies in acromegaly. The occurrence of protrusions of the internal carotid artery (ICA) (35–53%), a narrow intercarotid distance, and an asymmetrical course was described. In 13–18% of acromegalic patients, the presence of an intracerebral aneurysm could be reported (incidence in the general population:0.8–1.3%). The selected studies were however performed with a small patient sample (range:1–257).

We present a case report of a 57y/o male patient with anomalies of the ICA (“kissing carotid arteries”) harboring a GH-secreting adenoma, which was resected via an endoscopic endonasal approach.

**Conclusions:**

There is an association between acromegaly and endothelial dysfunction, which increases cardiovascular risk factors and vascular anomalies. Preoperative vascular imaging, e.g., CT angiography, should be implemented as a standard to identify patients at risk and estimate surgical morbidity. However, no evidence-based recommendations exist so far, so future studies are necessary.

**Supplementary Information:**

The online version contains supplementary material available at 10.1007/s11102-022-01291-3.

## Introduction

Acromegaly is a disorder characterized by overproduction of growth hormone (GH) and the resulting elevation of insulin-like growth factor (IGF-1). In over 95% of cases, the disease is caused by a benign pituitary adenoma of monoclonal origin [1, 2]. Acromegaly represents approximately 12% of all pituitary adenomas [3]. First-line therapy is the transsphenoidal resection, commonly by endoscopic guidance [1, 4]. The surgical treatment of an acromegalic patient with kissing carotid arteries in the presence of a pituitary adenoma prompted us to conduct the following literature review. This review addresses the surgical challenges posed by this group of patients.

On the one hand, the increased release of growth hormone through a pleiotropic effect results in increased morbidity due to cardiovascular disorders (arterial hypertension, systolic/ diastolic dysfunction, valvulopathies, cardiac arrhythmias, cardiomyopathy, myocardial infarction), cerebrovascular (stroke), metabolic disorders (diabetes mellitus, impaired glucose tolerance, and dyslipidemia) and respiratory disorders (obstructive sleep apnea syndrome, macroglossia, airway obstruction) [5–11]. Vascular endothelial dysfunction can be detected at the subclinical level (flow-mediated dilatation (FMD), carotid intima-media thickness, pulse-wave velocity, elastic modulus, epicardial adipose tissue thickness, the augmentation index[6, 12–15], as well. These pathophysiological conditions increase the risk of anesthesia and promote the occurrence of peri- and postoperative complications [16].

On the other hand, numerous studies[17–36] reported that anatomic vascular anomalies are associated with a higher risk of internal carotid artery injury during surgery. In such cases, life-threatening bleeding can occur, often challenging to manage using the endonasal endoscopic technique. Internal carotid artery injury during transsphenoidal surgery is reported in 0.1 to 0.9% of cases [37–39]. This condition is often lethal; however, no data on the exact mortality rate is available. Extrapolating from carotid blow-out syndrome, expected mortality should rise at least 30% [40].

Only few studies exist exploring systemic effects of GH and IGF-1 in patients with acromegaly. Sparse publications regarding anatomical vascular changes exist, most of them only presenting case reports. To our knowledge, no manuscript has explored these changes in acromegalic patients. Therefore, we performed a systematic literature review on vascular changes in patients with acromegaly to outline general comorbidities and highlight pitfalls concerning the surgical procedure.

## Methods

### Search strategy and inclusion criteria

A systematic review of the current literature was conducted by the Preferred Reporting Items for Systematic Reviews and Meta-Analyses (PRISMA) guidelines (Supplementary material). We searched the MEDLINE/ Pubmed database using the keywords ‘’acromegaly” combined with “endothelium”, “cardiovascular”, “vascular”, “aneurysm”, “dilatation”, “intercarotid distance”, and “kissing". This review follows the recommendations of the PRISMA statement. The databases were searched for articles published until July 2021. After entering the search mentioned above terms in EndNote X8 (Thompson Reuters, Carlsbad, California, USA), two authors (DCS and ESM) independently screened titles and abstracts, implementing those which met inclusion criteria. Disagreements were resolved through discussion. The search process could identify 567 records. After removing 226 duplicates, 258 abstracts were excluded because they were not written in English, a review, without full-text eligibility, or dealing with mean side effects of acromegaly independent of a pituitary adenoma (Supplementary material).

## Results

Following the selection process, we included 30 studies published between 1978 and 2020, which dealt with the following two issues: Twelve authors referred to systemic cardiovascular risk factors in patients with GH- and IGF-1-secreting adenoma. At the same time, sixteen studies reported vascular anomalies, mainly concerning the internal carotid arteries.

### Illustrative case

A 57-year-old male patient presented to our clinic with typical acromegaly characteristics, coarsening facial features, and enlargement of hands and feet. Neither cardiovascular risk factors nor systematic cardiovascular diseases could be diagnosed in preoperative cardiological examination. MRI and CT angiography revealed the presence of kissing internal carotid arteries with contact in the intracavernous space. Both, the concentration of GH with 15 ng/ml (reference value: < 6 ng/ml) and the level of IGF-1 with 981 ng/ml (reference value: 48–209 ng/ml) were preoperatively increased. An endoscopic transnasal transsphenoidal approach was used for total tumor resection. With the support of a neuronavigation system (BrainLab) with MRI and CT angiography data, the course of the internal carotid arteries could be outlined. Immunostaining of tumor tissue collected intraoperatively showed GH- and prolactin-producing cells, leading to the diagnosis of an GH- and prolactin-producing pituitary adenoma. Directly after tumor resection, IGF-1- and GH-levels have already decreased (GH: 4 ng/ml; IGF-1: 237 ng/ml) and were finally normal nine months after surgery (GH: 1 ng/ml; IGF-1: 215 ng/ml). The last follow-up three years after surgery demonstrated consistently normal IGF-1 and GH-values.

## Discussion

As broadly described in the literature and implemented in the diagnostic workup and treatment concepts of acromegalic patients, systemic diseases affecting, among others, the cardiovascular system occur due to GH over-secretion [1]. To determine the causes of the pathological changes, some authors present subclinical changes in the vessels based on ultrasound diagnostics [6–12]. In addition to these risk factors for the course of anesthesia, the anatomic vascular variations of the internal carotid artery pose particular challenges to the neurosurgeon for intraoperative management.

### Anatomic vascular anomalies

Vascular anomalies of the cerebral arterial circle (Circle of Willis), visualized by CT and MR angiography, were reported in 22 of the included studies[17–36] (Table 1). The occurrence of aneurysms in patients with elevated GH levels was described in 15 studies[17–28, 35, 36], however, with ten representing single case reports [17, 18, 20–28]. Oshino et al. [19] referred to 208 patients, nine of whom had aneurysms (4%). Manara et al. [26] included 152 patients with 28 aneurysms (18%), Pant et al. [28] reported five cases of aneurysms in 119 patients (4%), Oh et al. [35] observed six patients with aneurysms in a patient cohort of 257 (2%) and Jakubowski et al. [36] described four cases of patients with acromegaly and the occurrence of an aneurysm. The prevalence of aneurysms in the acromegaly samples presented in these studies is close to the prevalence in the general population. According to recent estimates, the prevalence of an unruptured cerebral aneurysm is 3.2% [41]. In this study, the authors note that the prevalence of an aneurysm in patients without comorbidities is double that of patients with a pituitary adenoma. This might suggest that patients with a pituitary adenoma have a lower risk of developing an aneurysm than the general population. The data from this review indicate that Acromegaly's prevalence is likely similar to the general population, given the additional cardiovascular risk factors associated with the disease. A narrowed distance between the internal carotid arteries (i.e., reduced intercarotid distance) was described in six studies [29–31, 33, 34, 42]. Three studies [29, 30, 32] described the appearance of carotid artery protrusion, and two studies [29, 30] noticed the loss of bony covering of the carotid artery in the cavernous segment. Table 2 elucidates that the expression of changes of the ICA is increased in acromegalic patients compared with the average population and even with patients with non-growth hormone-producing pituitary adenomas.

**Table 1:** Vascular anatomic characteristics in acromegalic patients caused by a functional pituitary adenoma; studies with the occurrence of a) aneurysms b) carotid artery protrusion c) a narrowed intercarotid distance between the cavernous portions d) a carotid artery dehiscence of canalis caroticus

(a) Aneurysms						
Author	n	Age (years)	Sex	GH-level (mean)	Location	Diameter (mm)
Zatelli et al. [17]	1	61	m	20.3 μg/l	Right MCA (distal + bifurcation)	24 mm
Sade et al. [18]	1	39	f	23.8 μg/l	Cavernous segment of the left ICA	6 mm
Oshino et al. [19]	208 (9 aneurysms)	Median 49	m: 101 f: 107	29 ng/ ml	ICA (5), ACA (3), MCA (1), BA (1)	Mean 6 mm (range:3–24 mm)
Holt et al. [20]	1	57	m	238 mU/l	Cavernous ICA	12 mm
Xia et al. [21]	1	48	f	2.4 ng/ ml	Cavernous ICA	4 mm
Seda et al. [22]	1	58	f	8.1 ng/ml	Supraclinoid ICA	12 mm
Curto et al. [23]	1	61	f	56 ng/ml	Cavernous ICA	12 mm
Acqui et al. [24]	1	38	f	59 ng/ml	Ophthalmic ICA	n.a
Hori et al. [25]	1	60	f	16.5 ng/ml	Cavernous ICA	5 mm
Manara et al. [26]	152 (28 aneurysm)	median 57 (59)	m: 70 (9) f: 82 (19)	35.9 μg/l (57.5)	ICA, MCA, ACA	Mean 3.5 mm
Weir et al. [27]	1	35	m	39 μg/l	Bilateral cavernous ICA	n.a
Pant et al. [28]	119 (5 aneurysm)	mean 42.5	m: 3 f: 2	n.a	4 PCA, 1 Ophthalmic ICA	Mean 3 mm (range: 3-4 mm)
Acqui et al. [24]	1	38	f	59 ng/ml	Ophthalmic ICA	n.a
Oh et al. [35]	257 (6 aneurysm)	mean 50	f: 6	n.a	ICA (4), MCA, ACA	Mean 4.4 mm (range: 2.3-7 mm)
Jakubowski et al. [36]	4	mean 50.5	m: 3 f: 1	n.a	Ophthalmic ICA, ACA, Cavernous ICA (2)	Mean 6 mm (range: 4-10 mm)

Carotid artery protrusion					
Author	n	Age (years)	Sex	GH-level	Protrusion (n)
Sasagawa et al. [29]	45	59 ± 10.8	m: 19 f: 26	n.a	16 (36%)
Sacher et al. [30]	1	74	m	n.a	1
Saeki et al. [32]	13	53.2	m: 7 f: 6	n.a	7 (53%)

Intercarotid distance (between cavernous portions)					
Author	n	Age (years)	Sex	GH-level	Intercarotid distance (n)
Sasagawa et al. [29]	45	59 ± 10.8	m:19 f:26	n.a	n.s
Sacher et al. [30]	1	74	m	n.a	1
Ebner et al. [31]	45	49 (range 9–80)	m: 21 f:24	n.a	1.64 ± 0.40 cm in the acromegalic patients and 1.90 ± 0.26 cm in the control group (P = .0005)
Yan et al. [33]	86	40 ± 10	m:46 f:40	40.6 ng/ml (range 11.4- 60.2 ng/ml)	Bilateral siphon carotid ectasias (25.5 ± 4.1 vs. 23.4 ± 3.5 mm, P = 0.001); bilateral lacerum segments (26.2 ± 3.2 vs. 24.1 ± 4.3 mm, P < 0.001)
Carrabba et al. [42]	23	50 (range 29–67)	m: 12 f: 11	n.a	A mean value of 14.83 mm in the GH group versus 16.65 mm in the nGH group (p = 0.05)
Mascarella et al. [34]	25	52.9 (± 14.3)	n.a	n.a	Clival segment compared with controls (1.59 cm and 1.77 cm; p = 0.02); paraclinoid segment compared with controls (1.50 cm and 1.33 cm; p = 0.01)

Carotid artery dehiscence					
Author	n	Age (years)	Sex	GH-level	Dehiscence (n)
Sasagawa et al. [29]	45	59 ± 10.8	m: 19 f: 26	n.a	10 (22%)
Sacher et al. [30]	1	74	m	n.a	1

**Table 2:** Vascular anomalies incidence in patients harboring GH-secreting and non-functioning pituitary adenomas, as well as in the average population

	GH-Secreting adenomas	Pituitary adenomas	Average population	Required radiological exam when discovered at CT/MRI	Preoperative precautions
Aneurysms (frequency)	4% [26]–18% [28]	2.3% [35]	2.1% [35]	DSA	Preoperative clipping/ Embolization discussion
Intercarotid Distance (cm)	Smaller at clival level (< 1.5 cm) [34]	Normal or increased in case of macroadenomas [34]	Clival: 1.76 Cavernous: 1.73 Paraclival: 1.33 [51]	None	Warn the anesthesiologist / prepare for muscle harvesting and nasal packing/Warn the neuro-radiologist
Carotid Protrusion	35% [29]	13.3% [29]	30% [52]	None	None
Dehiscent parasellar carotid bony canal	22% [29]	6.6% [29]	5% [52]	None	Warn the anesthesiologist/prepare for muscle harvesting and nasal packing/Warn the neuro-radiologist

### Systemic cardiovascular risk factors

Eleven studies [5–15] describe the development of cardiovascular risk factors caused by the increase in GH level and the remission of these cardiovascular risk factors after surgical resection of GH-producing pituitary adenoma and decreased GH concentration. Of these, seven authors [5–11] (Table 3) describe clinically measurable changes that can be determined using transthoracic echocardiography or laboratory chemical parameters. Five studies [5–8, 10] dealt with deterioration of left ventricular function and hypertrophy of the left ventricle under elevated levels of GH and IGF-1 and improvement of these parameters post-resection. The impact on glucose metabolism was reported in four studies [5, 7, 10, 11]. Reduction of preoperative hypertensive systolic blood pressure levels to normotensive levels after surgery has been addressed in four studies [5, 7, 8, 11]. Besides, Guo et al. [10] observed an improvement in right ventricular function and the decline of development of myocardial fibrosis postoperatively observed in the echocardiography. These patient samples ranged from n = 21 to n = 303.

**Table 3:** Systemic alterations due to increased secretion of growth hormone in patients with pituitary adenomas

Author	n	GM	Syst. BP	LV diastolic dysfunction	LV mass index	CAVI	RV systolic dysfunction	MF
Zhang et al. [5]	24	X	X	X	X			
Cansu et al. [6]	53			X	X			
Jaffrain-Rea et al. [7]	31	X	X	X	X			
Minniti et al. [8]	30		X	X	X			
Matsuda et al. [9]	21					X		
Guo et al. [10]	50	X		X	X		X	X
Altuntaş et al. [2019]	303	X	X					

*GM* glucose metabolism, *Syst. BP* systolic blood pressure, *LV* left ventricular, *CAVI* cardioankle vascular index, *RV* right ventricular, *MF* myocardial fibrosis

In five studies [6, 12–15] (Table 4), the effect of GH elevation on subclinical changes, including structural and functional arterial parameters, was investigated. Structural changes include carotid intima-media thickness and epicardial adipose tissue thickness. Functional parameters compromise flow-mediated vasodilation (FMD), pulse wave velocity, arterial stiffness measured by the augmentation index, and pressure-strain elastic modulus.

**Table 4:** Subclinical vascular structural (CIMT, EAT) and functional changes (FMD, PWV, EP, Aix) measured by ultrasonography of the ICA

Author	n	FMD	CIMT	PWV	EP	EAT	Aix
Ghiorghe et al. [12]	54	X					
Cansu et al. [6]	53		X	X			
Yaron et al. [13]	29	X					
Galoiu et al. [14]	64		X	X	X		X
Ozkan et al. [15]	79	X	X			X	

*FMD* flow mediated dilatation, *CIMT* carotid intima-media thickness, PWV pulse-wave velocity, *EP* elastic modulus, *EAT* epicardial adipose tissue thickness, *Aix* the augmentation index

Ozkan et al. [15] reported structural changes by measuring carotid intima-media thickness by ultrasound and the epicardial adipose tissue thickness in the right ventricle by echocardiography. In addition to Ozkan et al., two other studies [6, 14] demonstrated increased carotid intima-media thickness using sonography, a well-known decisive marker to quantify and evaluate carotid plaque formation that serves as a biomarker of subclinical atherosclerosis. Three authors [12, 13, 15] described impaired endothelial function measured by ultrasound based on decreased FMD. Galoui et al. [14] analyzed the Augmentation Index and elastic modulus as additional functional subclinical changes. Doppler sonography calculated the parameters that determined systolic and diastolic blood pressures measured in the internal carotid and brachial artery regions. Augmentation index as a correlate of vascular stiffness was significantly increased in the patients with elevated IGF-1 and GH levels compared with the control group with controlled acromegaly. An opposite trend could be observed concerning vascular elasticity, determined by elastic modulus. An increased pulse wave velocity in acromegalic patients was detected sonographically in two studies [6, 14].

### Clinical changes

First, an increased incidence of disorders in glucose metabolism is observed in patients with acromegaly. These include increased body mass index (BMI), blood glucose levels, triglycerides and LDL levels, hepatic gluconeogenesis and glycogenolysis, and hepatic and peripheral insulin resistance [5, 7, 10, 11, 43]. However, Zhang et al. [5] and Jaffrain-Rea et al. [7] showed that insulin resistance improves significantly after successful resection of growth hormone-secreting pituitary adenomas. Thus, surgical treatment is pivotal in reducing overall morbidity and mortality from this disease.

Secondly, pathological cardiovascular changes develop due to the increased secretion of growth hormone: In this group of patients, the occurrence of elevated systolic blood pressure, diastolic dysfunction with left ventricular hypertrophy, systolic dysfunction, the development of myocardial fibrosis, and an increased arterial stiffness as measured by the cardio-ankle vascular index (CAVI) can be observed. Similar to the metabolic changes, a remission of these pathological systemic changes is also apparent after tumor resection. For example, Zhang et al. demonstrated an improvement in blood pressure and echocardiographic evidence of a decreased LVM index and increased ejection fraction one year after surgery. The same conclusion was reached by others [7, 8]. Guo et al. [10] observed reversibility of cardiac alterations mainly in male patients but not all female patients. Matsuda et al. [9] observed a reduction in arterial stiffness as a marker for atherosclerotic changes by measuring CAVI in 15 patients with treated acromegaly. However, ten patients received systemic therapy with somatostatin analogues in addition to transsphenoidal surgery. A beneficial effect of somatostatin analogues treatment on reducing cardiovascular risk factors (e.g., left ventricular mass index, interventricular septum.

Thickness, left ventricular posterior wall thickness, the ratio of the E-wave and A-wave peak velocities of the mitral flow profile, ejection fraction and exercise duration) in acromegalic patients could be partially demonstrated [44]. However, the included patient cohorts are of a small case number and fail to show significant differences between medical treatment and microsurgical resection [45, 46].

### Subclinical changes

Using Doppler echocardiography of the internal carotid artery, subclinical parameters, which promote the development of atherosclerosis, were measured by some groups. These include structural (carotid intima-media thickness, epicardial adipose tissue thickness) and functional (pulse wave velocity, elastic modulus, epicardial adipose tissue thickness, augmentation index) parameters. Interestingly, when comparing two cohorts of patients with controlled and active acromegaly, Galoiu et al. [14] and Ozkan et al. [15] could not identify significant differences in clinically apparent cardiovascular risk factors (diabetes mellitus, dyslipidemia, arterial hypertension). In contrast, significant differences could be observed for subclinical structural and functional measures. After normalization of growth hormone levels, only an improvement in functional but not structural parameters was noticed by Galoiu et al. [14]. Cansu et al. [6] found a significant difference in clinically apparent factors (left ventricular function and mass index) and subclinical factors (carotid intima-media thickness, pulse wave velocity) between patients with and without acromegaly.

However, there was no difference between controlled and uncontrolled acromegalic patients. In addition, Yaron et al. [13] found no differences in pulse wave velocity, carotid intima-media thickness, and the augmentation index between treated and nontreated patients. Only differences in endothelial function measured by FMD were detected. The difference in FMD was also confirmed by Ghiorghe et al. [12]. Thus, the meaningfulness of subclinical parameters is highly controversial in the literature, and their value remains vague.

In conclusion, these parameters show that the arterial vessel walls in acromegalic patients are stiffer, thicker, and less elastic. This may be associated with a higher risk of carotid artery injury and raises the question of improving the arterial wall physiology to allow more aggressive safe resection of these tumors. In this context, the benefit of preoperative somatostatin analogues therapy may be useful to help normalize the cardiovascular risk profile prior to surgery. Furthermore, the question arises whether vessel wall imaging with MRI black blood sequences helps show inflammation of the ICA.

### Anatomic vascular anomalies

Due to the smooth muscle hypertrophy of the arterial walls and changes of the intima from the increased growth hormone secretion, altered vascular configurations develop. This may lead to dolochoectasia of the intracranial vessels and vascular protrusion. Aneurysms are most likely attributed to focal areas of abnormally-shaped vessels due to smooth muscle hypertrophy. In this context, dolochoectasia and protrusion are not equivalent to the presence of an aneurysm.

### Aneurysm

Oshino et al. [19] included a patient cohort of 208 acromegalic patients, and 9 (4.3%) showed intracranial aneurysms. In the control group of more than 7390 patients, a prevalence of 1.8% could be identified. Besides, they also reported a significant male predominance (7 of 9 patients). The mean diameter of the aneurysms was 6 mm (range: 3–24 mm). Likewise, Pant et al. [28] describe a prevalence of 4% for aneurysms in a group of 119 patients with elevated growth hormone levels. The highest overall prevalence of 25% was found in the group of patients with FSH-producing adenomas. However, it should be noted that only four patients were included in this group. To be sufficiently reliable about differences in vascular abnormalities between the different hormone expressions, the inclusion of a more significant number of patients in each group is required. A higher prevalence of acromegalic patients with aneurysms of 17.3% is found by Manara et al. [26].

Nevertheless, the colleagues showed a relatively high prevalence of aneurysms in the control group, with 7%. Notably, this study revealed a significant difference between the growth hormone level and aneurysms' occurrence. Most studies present case reports of coincidence of pituitary adenomas with aneurysms. The GH level is only partly comparable with different units and reference values. The localization, as well as the extent of the aneurysms, is depicted in Table 1. Acqui et al. [24] examined collagen composition in a patient with a GH-producing pituitary adenoma and the coincident finding of an ICA aneurysm in the ophthalmic segment. It determined a deficiency of type III collagen. Investigating a larger cohort of patients is needed to assess the role of collagen expression.

### Carotid artery protrusion

Another vascular anomaly relevant to the transsphenoidal approach is a protrusion of the internal carotid artery in the cavernous segment. This is attributed to the general bony changes in acromegalic patients and is defined as "half or more cross-sectional area of carotid artery protruded into sphenoid sinus space on axial view". For this purpose, CT scans of 1 mm slice thickness of 45 patients with acromegaly were compared with a control group of equal size. A significantly higher incidence in the acromegalic patients was noted (35.5% vs. 13.3%; p = 0.013) [29]. Similarly, Saeki et al. [32] reported an increased incidence of protrusion of the internal carotid artery with 53.4% (7/13) in acromegalic patients compared to 18.25% (8/44) in a sample of patients with non-GH secreting pituitary adenomas. However, carotid prominence is not precisely defined in this study. Sacher et al. [30] present a case report of a patient with kissing ICAs, comparable to the patient treated in our hospital. Axial CT imaging depicted dilatation in the cavernous segment with only a minimal gap between the arteries.

### Intercarotid distance (between cavernous carotid arteries)

Sasagawa et al. [29] could not find a significant difference concerning the intercarotid distance or Knosp grade. [47] Likewise, Yan et al. [33] could not observe a significant correlation between Knosp grade and acromegaly. The Knosp grade defines the cavernous sinus invasion of the tumor and was most notably not referred to for comparison by others, although this grading is used in clinical practice [48]. The intercarotid distance was measured as the smallest distance between the medial walls of the intracavernous portion of the cavernous carotid arteries on axial CT slices. In contrast, Ebner et al. [31] and Yan et al. [33] found a significant narrowing of the intercarotid distance in patients with acromegaly and pituitary tumors. For this purpose, Ebner et al. [31] examined 45 acromegalic patients. They measured the ICA bony canal level at the beginning of the presellar segment using thin-slice axial CT slices. They determined the distance between the inner walls on the one hand and the outer walls of the arteries on the other. Interestingly, there was only a significant difference in the measurements between the inner wall (1.64 ± 0.40 cm in the acromegalic patients and 1.90 ± 0.26 cm in the control group (p = 0.0005). In addition, a significantly larger diameter of the ICA in this segment was noticed in the acromegalic patients. A detailed measurement of the distance between the inner walls of the ICA at the level of ophthalmic, siphon carotid ectasias, cavernous and lacerum segments was performed by Yan et al. [33] For this purpose, the colleagues used a three-dimensional software. They found significant differences in the distance between siphon carotid ectasias and bilateral lacerum segments, but there was no significant difference between the ophthalmic and cavernous segments. The bony alterations in acromegalic patients, which become apparent in the form of coarsening facial features and enlargement of the extremities, can thus be observed in the skull base. These result in anatomic alterations and, therefore, more demanding planning of the transsphenoidal approach [49]. Carrabba et al. [42] reported in a cohort of 23 acromegalic patients with pituitary adenoma, compared to a control group without GH excess, a reduced intercarotid distance, and a deeper sphenoid bone and more intrasphenoid septa. To our knowledge, no correlation was found between the anatomical anomalies and the level of GH or IGF-1, [31, 33]. In contrast, a significant correlation between the duration of the disease and the anatomical alterations could be noticed [33].

### Carotid artery dehiscence

Carotid artery dehiscence was determined by Sasagawa et al. [29] when there was no bony layer around the ICA at the carotid canal wall on the axial CT slices. The authors observed a significant increase in dehiscence in acromegalic patients (22.2% vs. 6.6%; p = 0.035).

In summary, there is a higher intraoperative risk of life-threatening intraoperative injury to the ICA due to the anomalies of the carotid artery and the alterations of the skull base landmarks. Meyer et al. [37] reported a case of a 60-year-old patient with acromegaly who had an accidental carotid artery injury during transsphenoidal tumor resection. To perform safer maximal resection of these tumors and ensure higher remission rates in this group of patients, it will be essential to assess the indications for preoperative planning with CT angiography, the use of intraoperative use of Doppler ultrasound, and when to perform cerebral digital subtraction angiography if injury or aneurysm is suspected. Giant pituitary adenomas, in particular, present a surgical challenge due to the extent of extension and infiltration into adjacent structures such as the cavernous sinus and ICA [50]. While reversibility of cardiovascular and metabolic impairment after successful therapy has been demonstrated [5–11], no study has addressed the reversibility of intracranial alterations (Table 2) .

## Limitations

In addition to GH levels, cardiovascular changes are attributable to the patient's lifestyle and thus relevant risk factors such as smoking, low levels of exercise, genetics, and obesity. Moreover, the number of included cases is limited to a small group of patients; this refers to the analysis of systemic comorbidities (n = 21–79) and anatomic vascular changes (n = 1–208). Some patients received radiation therapy or intake of somatostatin analogues in addition to surgery.

## Conclusions

**Fig. 1:**
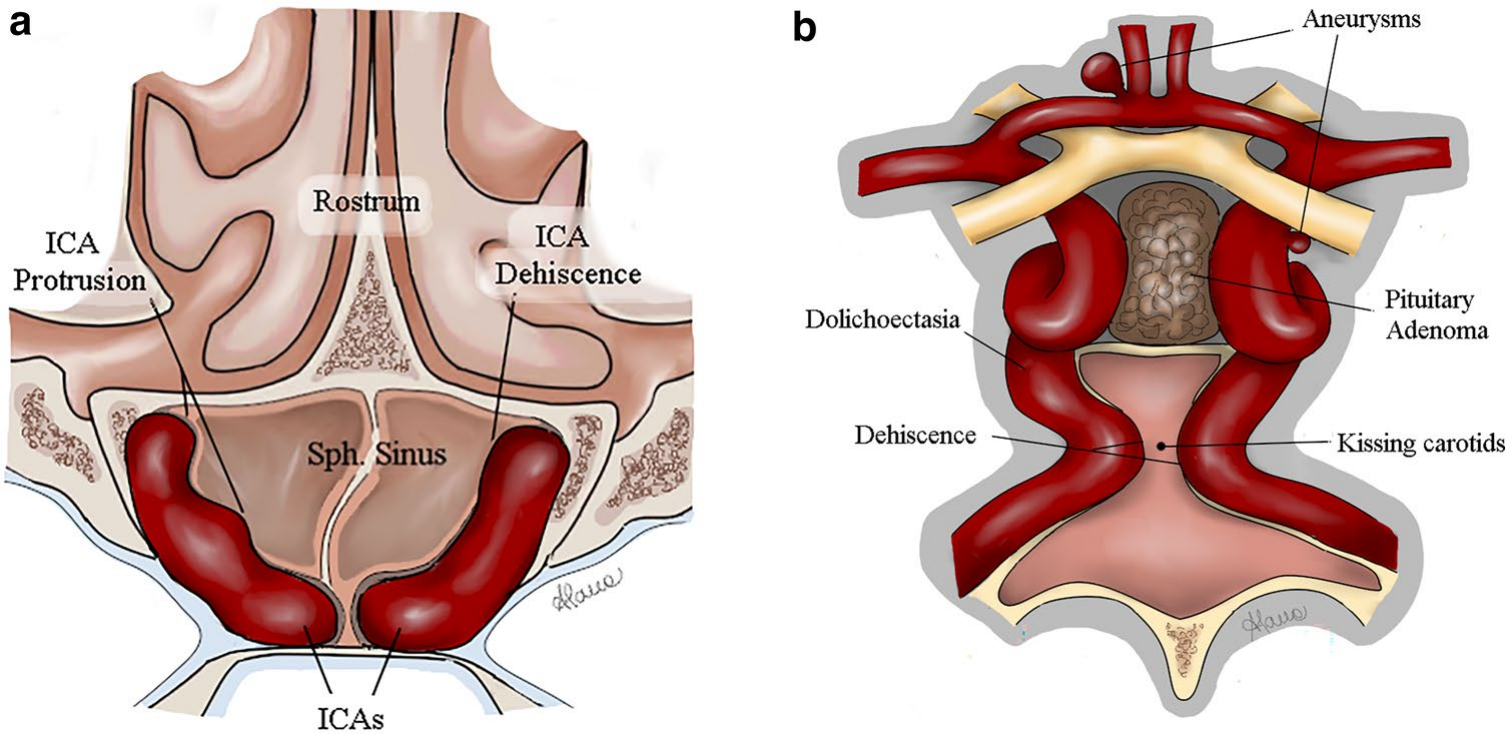
Schematic figures of vascular anomalies, including aneurysms, carotid artery protrusion, dehiscence, and a narrow distance between the cavernous segment in **a** axial and **b** coronal reconstruction

**Fig. 2:**
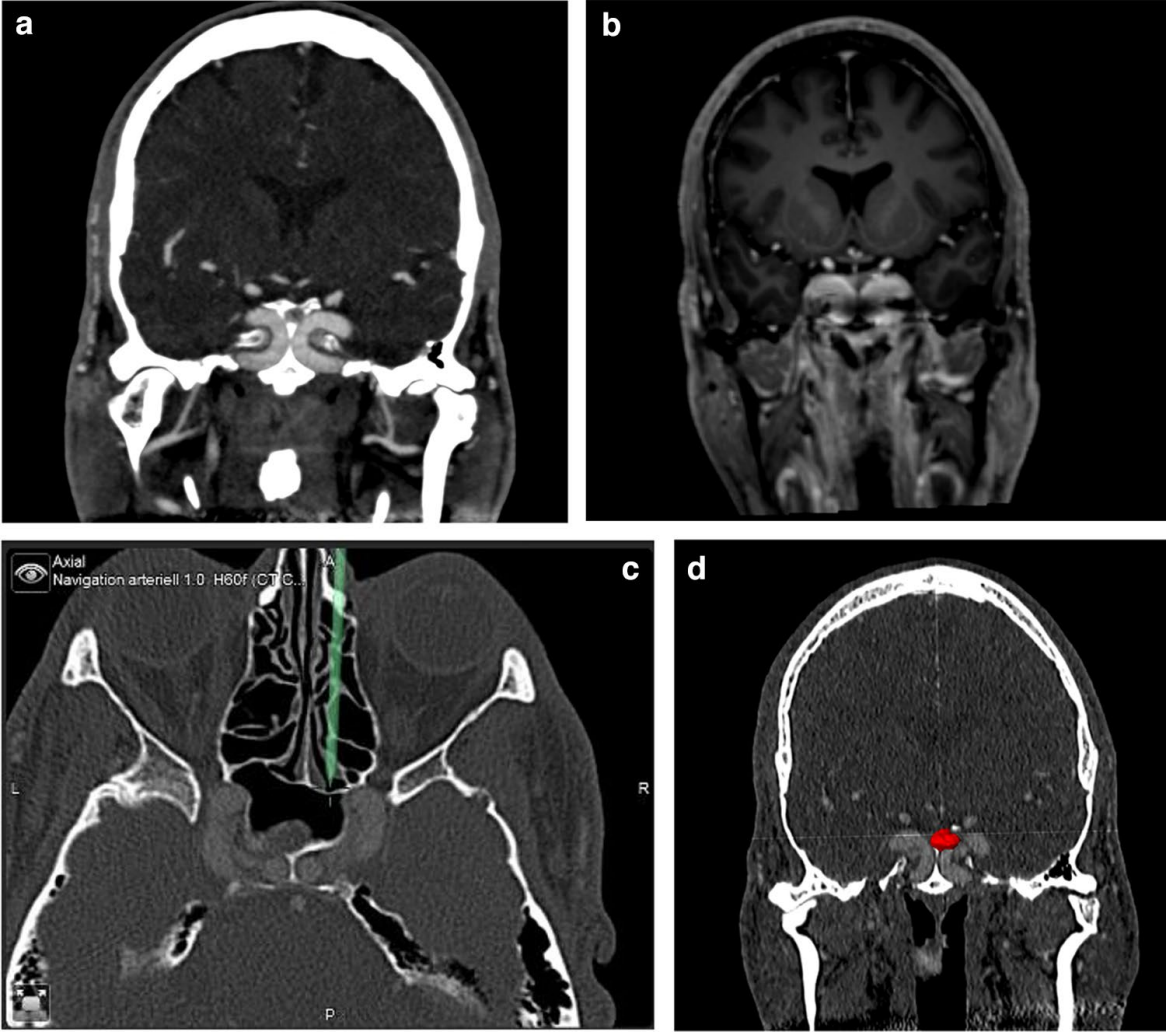
MRI and CT angiography demonstrating **a** a 57-year-old male acromegalic patient with **a** narrow intercarotid distance at paraclival and intercavernous space (kissing carotid arteries), **b** MRI angiography demonstrating the poor spatial resolution in this region, **c** the protrusion of the carotid artery into the sphenoid sinus with an extension up to the rostrum of the cavernous sinus and **d** CT angiography with marked STH-producing microadenoma

Neurosurgical patients with acromegaly should be screened for systemic cardiovascular risk factors. These carry an increased risk of anesthesia and a generally increased postoperative morbidity. Furthermore, the prevalence of vascular anomalies is higher in acromegalic patients than in the general population. Adequate vascular imaging with CT- or MRI angiography seems to increase surgical safety in these patients. An additional low-threshold indication for DSA should be considered if an aneurysm is suspected. ICAs' shape, size, and course should be carefully studied before surgery. In case of anomalies, a neuro-radiologist team should be held on standby. For further clarification, conducting a prospective, multicenter study is recommended to characterize this patient population’s vascular anomalies and assess the cost-effectiveness of such interventions before making them the standard of care (Figs. 1 and 2).

## Supplementary Information

Below is the link to the electronic supplementary material.

Supplementary file1 (PPTX 64 kb)

## Data Availability

Not applicable.
