# Supplementary material for: Vascular anomalies in patients with growth hormone-secreting pituitary adenomas: illustrative case report and systematic review of the literature
Source: Pituitary. 2022 Dec 12;26(1):132–43. doi: 10.1007/s11102-022-01291-3 (PMC9908726; doi:10.1007/s11102-022-01291-3)
Supplement: Supplementary file 1 — Supplementary file1 (PPTX 64 kb) [file 11102_2022_1291_MOESM1_ESM.pptx]

## Slide 1
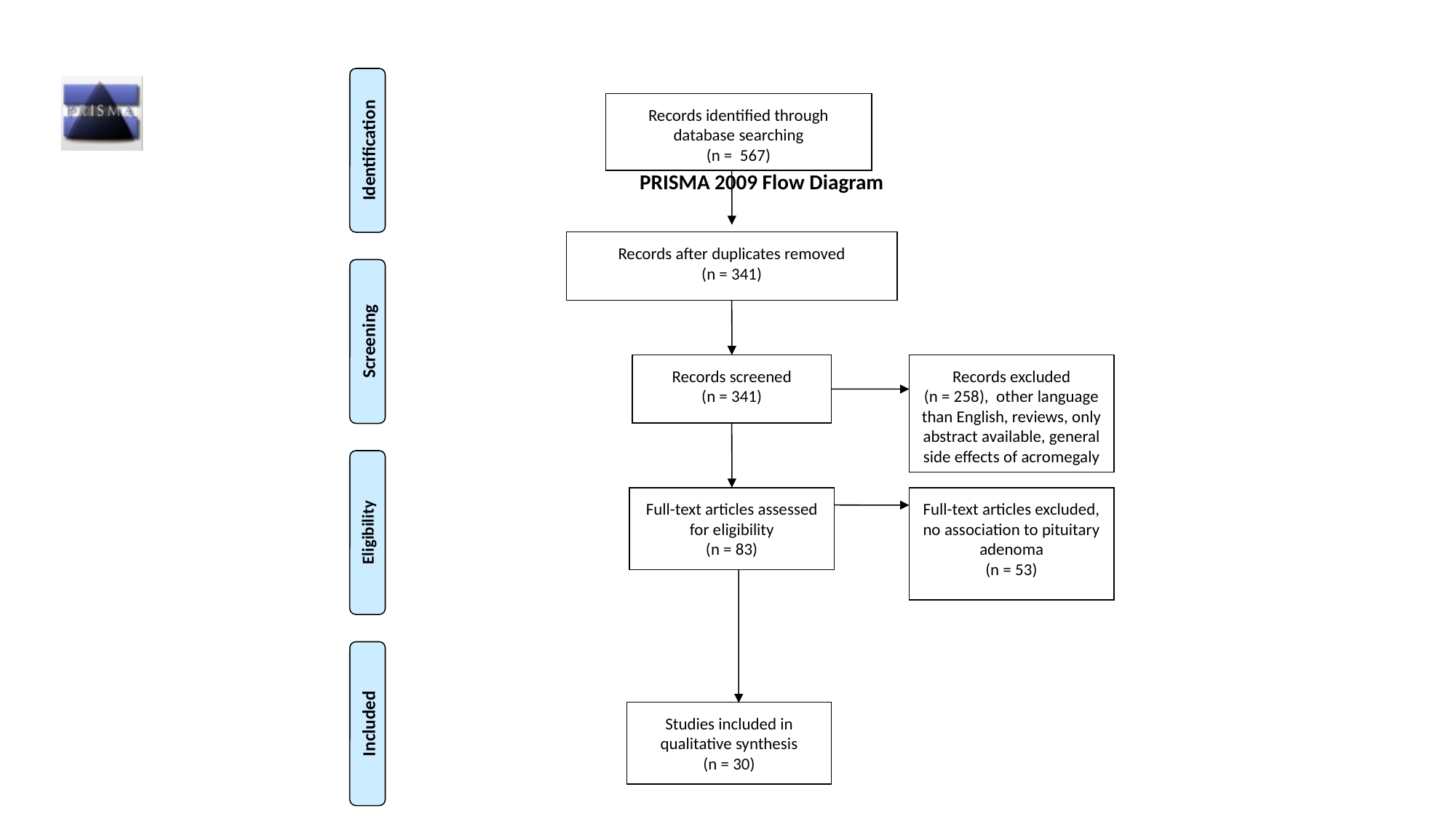

Records identified through database searching
(n = 567)
Identification
PRISMA 2009 Flow Diagram
Records after duplicates removed
(n = 341)
Screening
Records excluded
(n = 258), other language than English, reviews, only abstract available, general side effects of acromegaly
Records screened
(n = 341)
Full-text articles assessed for eligibility
(n = 83)
Full-text articles excluded, no association to pituitary adenoma
(n = 53)
Eligibility
Studies included in qualitative synthesis
(n = 30)
Included
